# Maternal rheumatoid arthritis and the risk of offspring autism spectrum disorder: two national birth cohorts and a meta-analysis

**DOI:** 10.1186/s13229-025-00694-w

**Published:** 2025-12-04

**Authors:** Evora Hailin Zhu, Benjamin HK Yip, Caroline Fyfe, Eugene Merzon, Arad Kodesh, Johan Askling, Abraham Reichenberg, Weiyao Yin, Stephen Z. Levine, Sven Sandin

**Affiliations:** 1 Jockey Club School of Public Health and Primary Care, The Chinese University of Hong Kong, Hong Kong SAR, China; 2 Department of Medical Epidemiology and Biostatistics, Karolinska Institutet, Stockholm, Sweden; 3 Leumit Health Services, Tel Aviv, Israel; 4 Adelson School of Medicine, Ariel University, Ariel, Israel; 5 Meuhedet Health Services, Mental Health, Tel Aviv, Israel; 6 Department of Community Mental Health, University of Haifa, Haifa, Israel; 7 Clinical Epidemiology Division, Department of Medicine Solna, Karolinska Institutet, Stockholm, Sweden; 8 Rheumatology, Theme Inflammation and Ageing, Karolinska University Hospital, Stockholm, Sweden; 9 Department of Psychiatry, Icahn School of Medicine, Mount Sinai, New York, USA; 10 Seaver Autism Center for Research and Treatment at Mount Sinai, New York, USA; 11 Department of Obstetrics and Gynecology, West China Second University Hospital, Sichuan University, Chengdu, China; 12 School of Public Health, University of Haifa, Haifa, Israel

**Keywords:** Autism spectrum disorder, Autoimmune, Cohort, Joint pain, Rheumatoid arthritis, Prenatal risk

## Abstract

**Background:**

Prenatal maternal rheumatoid arthritis (RA) is postulated to increase the risk of offspring with Autism Spectrum Disorder (ASD), yet findings of the association are inconsistent, potentially owing to small sample sizes and insufficient consideration of the timing of RA onset. This study aimed to examine the association between maternal RA, particularly its timing, and offspring ASD risk.

**Methods:**

Two Israeli birth cohorts with national coverage and a meta-analysis were analyzed. Two harmonized birth cohorts of individuals born between 2003 and 2014 from Israeli Health Maintenance Organizations were followed up to 2021–05-01. Meta-analysis included all studies published up to 2023–06-07. Two exposures differentiated the timing of RA onset through maternal RA diagnosed before or after delivery. The association between maternal RA and the risk of offspring ASD was quantified by separate and pooled hazard ratios (HRs) from Cox proportional hazards regression with adjusted confounders. In the meta-analysis, the association was quantified using odds ratios (OR) fitted with random effects models with sensitivity analyses testing heterogeneity.

**Results:**

Two Israeli birth cohorts included Cohort I (Population = 251,903; ASD = 1,939 [0.77%]) and Cohort II (Population = 309,696; ASD = 3,008 [0.97%]), contributing a total of 5.9 million person-years. Meta-analysis included 13 eligible studies (9 eligible estimates, population = 4,015,055, ASD = 45,124). Maternal RA before delivery increased the risk of offspring ASD (Cohort I HR = 1.55, 95% CI = 0.98–2.46; Cohort II HR = 1.83, 95% CI = 1.30–2.58; Combined I + II HR = 1.77, 95% CI = 1.45–2.17. Meta-analysis: OR = 1.57, 95% CI = 1.31–1.87).

**Limitations:**

Very few cases of seronegative RA compared to seropositive cases; RA subtype analysis was not feasible. It is important to acknowledge that the inclusion of the exposure “RA after delivery” also encompasses future RA information after the diagnosis of ASD, which may introduce biases. The association between RA drugs and offspring ASD risk has not been established due to a lack of data.

**Conclusions:**

Drawing on the strengths of two parallel birth cohorts from Israel and supported by meta-analysis, the current study indicates a modest but robust increase in the risk of ASD among offspring of mothers diagnosed with RA before delivery, but not when diagnosed only after delivery. This temporal specificity argues against shared genetic etiology and points toward maternal inflammatory status during pregnancy as a causal factor in offspring ASD risk.

**Supplementary Information:**

The online version contains supplementary material available at 10.1186/s13229-025-00694-w.

## Introduction

Autism spectrum disorders (ASDs) are neurodevelopmental disorders characterized by impairments in reciprocal social interaction, communication, and stereotyped repetitive behaviors and/or highly restricted interests [1]. The etiology of ASD remains unclear. Multiple studies substantiate the contributions of genetic [2–4] and prenatal factors to ASD [5]. One prenatal factor that is postulated to be linked to the risk of ASD in offspring is maternal rheumatoid arthritis (RA). RA is a chronic autoimmune inflammatory disorder affecting approximately 0.5%–1% of the population worldwide [6], and in France, approximately 0.1% of pregnant women [7]. Several lines of evidence point to an association between maternal RA and offspring ASD.

ASD is a condition that is known to run in families. Plausibly, familial autoimmunity and ASD may be associated, but the common etiology remains uncertain [8]. Multiple studies indicate that there are genes common to both ASD and RA, which support the hypothesis that there is an association between the two diseases [9–12]. Prenatal environmental factors may also contribute to the association, given that the presence of autoantibodies is a defining feature of the seropositive subtype, the most common form of RA [13]. The mother-to-child transmission of autoantibodies has been postulated to impair fetal brain development at the protein level, thereby increasing the risk of ASD [14, 15]. Therefore, delivery timing relative to maternal RA is relevant to differentiate heritable and non-heritable contributions of RA to the development of ASD in offspring. To date, the only large population-based study to appropriately examine timing found that maternal RA before, not after, delivery was associated with an increased risk of offspring ASD [16].

Existing epidemiological studies of the association between maternal RA and the risk of ASD in the offspring are rare and generally underpowered because of the combination of rare outcomes and rare exposures [17–20]. Meta-analyses have yielded inconsistent results; they were based on a few eligible studies, overlapping populations, and were unable to address the timing of maternal RA relative to offspring delivery [21–23]. Hence, better-informed evidence is required to delineate the association between maternal RA and the risk of ASD in the offspring.

The current study aims to test the hypothesis that maternal RA, particularly its timing before delivery, is associated with an increased risk of offspring ASD based on two national cohorts and a meta-analysis.

## Methods

### Study 1: cohort studies

#### Study design and population

A cohort study design was implemented using two Israeli national birth cohorts of children. In Israel, medical insurance is compulsory for every de jure resident. Since 1995, the National Health Insurance Law has mandated all residents, to join one of four non-profit health maintenance organizations (HMOs). It is illegal for non-profit HMOs in Israel to refuse membership based on demographic factors, geographical location, health conditions, or medication needs, thereby limiting sample selection bias in this study. The current study source population was two cohorts of children born alive between 2003 and 2014, and derived from two HMOs (Cohort I and Cohort II, hereafter for anonymity). These two HMOs provide health care to over 2 million members in total. In Israel, each de jure resident has a unique identification number assigned at birth or immigration, which allows maternal-offspring data linkage. Encrypted identification numbers were received. Ethical approval to undertake the study was granted by the Institutional Review Boards of each HMO and the University of Haifa, in accordance with the Declaration of Helsinki, with a waiver of informed consent as data were already anonymized. Uncovering participants to request informed consent was therefore not possible. This study followed the Strengthening the Reporting of Observational Studies in Epidemiology (STROBE) reporting guideline [24].

#### ASD ascertainment

In Israel, children with a clinical impression of possible ASD undergo evaluation by an expert panel comprising social workers, a psychologist, and a medical board-recognized psychiatrist, developmental-behavioral pediatrician, or child neurologist. A board-recognized developmental-behavioral pediatrician determines the final ASD diagnosis. ASD diagnosis was ascertained using the 9th and 10th versions of the International Classification of Diseases (ICD-9 and 10; for codes, see Supplement eTable 1) since 1997. Diagnoses of maternal RA and offspring ASD were followed up to May 11, 2021. Children with an ASD diagnosis after age one and before the end of follow-up were classified as having ASD.

#### Ascertainment of maternal RA

RA assessment for each de jure resident is free and needs-based. Maternal RA was classified based on diagnoses by the HMO’s rheumatologist (see Supplement eTable 1). Pre-delivery RA was defined as any diagnosis before and within 90 days after delivery to adjust for delayed RA diagnosis ascertainment; post-delivery RA was defined as any diagnosis more than 90 days after delivery until the end of follow-up.

#### Covariates

Generally, maternal age at delivery is older among mothers with RA and ASD compared to the general population and so was therefore included as a confounder. Both RA and ASD prevalences have increased by calendar year, so the year of delivery was included as a confounder. Information on maternal age at delivery (years), biological sex (male/female), and year of delivery was obtained from the HMOs.

#### Statistical analysis

The two birth cohorts were analyzed separately, and pooled risk estimates were derived from the weighted cohort estimates. Each child was followed up from delivery until their first ASD diagnosis, death, or end of follow-up on May 11, 2021, whichever came first. The risk of offspring ASD was quantified by fitting hazard ratios (HRs) from Cox proportional hazards regression models with age as the underlying time scale and with associated two-sided 95% confidence intervals (CIs). The assumption of proportional hazards was examined by visual inspection of the weighted Schoenfeld residuals [25]. We also reported approximated pooled risk HRs assuming a Gaussian distribution [26]. Robust standard errors (“sandwich estimators”) were calculated using the clusters of maternal identification numbers to adjust for potential within-family correlations [27].

Two models of sequentially increased adjustment were computed to identify potential sources of confounding. Model 1 was adjusted for birth year using natural splines with degrees of freedom set to 3 [28]. Model 2 supplemented Model 1 with additional adjustments for offspring sex and the maternal age at delivery in splines (degrees of freedom = 3). Both models were fitted separately for both maternal RA exposures, including “RA before delivery” and “RA after delivery”. All tests of statistical analyses were conducted at the two-sided 5% level of significance without adjustment for multiple statistical tests.

#### Complementary analyses

Additional models were fitted to directly compare RA diagnosed before delivery versus RA with onset after delivery, among all mothers with RA diagnoses, regardless of timing. One sensitivity analysis was implemented, based on prior studies [22], without specifying the timing of maternal RA related to offspring delivery to test the robustness of the association.

### Study 2: meta-analysis of maternal RA and ASD in offspring

A meta-analysis was implemented to examine the association between maternal RA and the risk of offspring ASD. RA exposures included RA before delivery and RA after delivery, compared to mothers without an RA diagnosis. The literature search was performed on PubMed, Embase, Web of Science, and Medline. This meta-analysis was reported in accordance with the Preferred Reporting Items for Systematic Reviews and Meta-Analyses (PRISMA) [29].

#### Search strategy

This meta-analysis search for studies published prior to June 7, 2023, aimed to examine the association between maternal RA and ASD in offspring. We used the Cochrane “PICO” framework [30]—patient/problem, intervention, comparison, and outcome—to define search terms related to maternal RA and offspring ASD. The complete search strategy is presented in eTable 1. A study was eligible for the meta-analysis if it was original research (not a case report, case series, or conference abstract), included a comparison group, and reported the risk estimate with adjustments for potential confounding factors.

#### Study selection, data collection, and quality assessment

The full-text literature screening was scrutinized for relevance by two independent reviewers (authors: EHZ and CF). Exclusions and inclusions are presented in eFigure 2. The results from the two Israeli cohorts described in this manuscript were also included. The Newcastle-Ottawa Scale assessed the data quality of the eligible studies, with higher values indicating higher quality [31]. In the case of studies with overlapping study populations (e.g., from the same country or area), to ensure independence between the studies, we retained only the study with the largest sample size and broadest birth cohort coverage.

#### Statistical analysis

Extracted studies were recorded with the author name, publication year, cohort name with follow-up period, country or area where the study was conducted, number of children, number of ASD cases, number of children with RA mothers, ascertainment method of identifying ASD and RA, RA exposure timing (before or after delivery), effect size, corresponding adjustments, and an indicator of whether or not it was included in the meta-analysis with justification.

We estimated crude odds ratios (OR), that is, models including only maternal RA status as a covariate, and adjusted ORs using random-effect models with 95% confidence intervals. We fitted random-effect models separately for “RA before delivery” and “RA after delivery”. Study heterogeneity was assessed with the I-squared statistic (0–40%, might not be important; 30–60%, might represent moderate heterogeneity; 50–90%, might represent substantial heterogeneity; 75–100%, considerable heterogeneity), and tau-squared statistics [32]. Publication bias was inspected using funnel plots.

#### Patient and public involvement

No patients were involved in setting the research question or the outcome measures, nor were they involved in the design and implementation of the study.

#### Sensitivity analysis

Three sensitivity analyses were conducted. The first involved cumulatively adding each study to the meta-analysis. The second was a leave-one-out analysis, in which one study was repetitively removed to evaluate if any single study caused heterogeneity. Third, a sensitivity analysis was conducted on the studies focusing on those that did not specify the timing of RA. This analysis aimed to assess the robustness of the association between maternal RA and offspring ASD.

## Results

### Study 1: cohort studies

#### Maternal RA and the risk of ASD in the offspring based on Maternal RA and offspring ASD maternal RA and offspring ASD: cohort I

A total of 14,624 of 266,551 (5.49%) children born between 2003 and 2014 were excluded from Cohort I for leaving the Cohort I system, death, or an ASD diagnosis before age one. This was done to ensure the accuracy of the ASD diagnosis. In the follow-up period of 2,456,729 person-years, the cohort finally comprised 251,903 children. (eFig. 1). There was no support in the data contradicting the assumption of proportional hazards after inspection of weighted Schoenfeld residuals. There were 20 of 1,408 (1.42%) ASD diagnoses among children born to mothers with RA before delivery and 1,919 of 250,495 (0.77%) ASD diagnoses among children born to mothers free from RA before delivery. Compared to offspring of mothers without RA before delivery, children born to mothers with RA before delivery had a slightly higher proportion of males and a higher maternal age at delivery for children born to mothers with RA during pregnancy (Table 1).

**Table 1:** Subject characteristics in the two birth cohorts

	Cohort I		Cohort II	
	Mothers with RA Number of children (%)	Mothers without RA Number of children (%)	Mother with RA Number of children (%)	Mother without RA Number of children (%)
Number of Children	1,408	250,495	1,717	307,979
Offspring sex, males (%)	759 (53.91%)	129,115 (51.54%)	893 (52.01%)	157,625 (51.18%)
ASD cases (%, person year)	20 (1.42%, 17,103)	1,919 (0.77%, 2,439,626)	33 (1.92%, 22,545)	2,975 (0.97%, 3,404,054)
ASD rate (cases per 100 000 person years)	117	79	146	87
Follow-up years (Median, Q1–Q3)	12.8 (9.2 - 15.5)	9.6 (6.7 - 13.2)	13.7 (10.6 − 16.3)	11.0 (8.0 − 14.4)
Birth year (%; Median, Q1–Q3):				
2003–2005	16.2 (14.4 - 17.3)	15.4 (8.3 - 16.7)	16.8 (16.0 − 17.6)	16.5 (15.6 − 17.4)
2006–2008	13.3 (11.8 - 14.4)	12.7 (8.6 - 14.0)	13.7 (12.9 − 14.5)	13.4 (12.5 − 14.4)
2009–2011	10.4 (9.5 - 11.3)	9.9 (7.0 - 11.1)	10.8 (10.0 − 11.6)	10.4 (9.5 − 11.4)
2012–2014	7.5 (6.7 - 8.4)	7.1 (5.4 - 8.2)	7.8 (6.9 − 8.7)	7.5 (6.6 − 8.4)
Children’s age at 1st ASD diagnosis, Median (Q1–Q3)	4.8 (2.7 - 8.7)	5.1 (3.1 - 7.8)	6.9 (4.4 − 10.0)	6.3 (3.9 − 9.2)
Birth year (%)				
2003–2005	528 (37.50%)	62,802 (25.07%)	651 (37.91%)	69,111 (22.44%)
2006–2008	426 (30.26%)	63,390 (25.31%)	499 (29.06%)	76,405 (24.81%)
2009–2011	269 (19.11%)	63,525 (25.36%)	351 (20.44%)	82,889 (26.91%)
2012–2014	185 (13.14%)	60,778 (24.26%)	216 (12.58%)	79,574 (25.84%)
Maternal age at delivery				
< 20	20 (1.42%)	6,351 (2.54%)	32 (1.86%)	7,320 (2.38%)
20–29	564 (40.06%)	139,867 (55.84%)	657 (38.29%)	147,321 (47.84%)
30–39	699 (49.64%)	93,610 (37.37%)	852 (49.65%)	136,212 (44.24%)
40–49	124 (8.81%)	10,476 (4.18%)	169 (9.85%)	16,921 (5.50%)
≥50	1 (0.07%)	191 (0.08%)	6 (0.35%)	153 (0.05%)

1. ASD: Autism Spectrum Disorder; RA: Rheumatoid Arthritis; Maternal RA defined as before delivery or within 3 months after delivery. Q1: First quartile; Q3: Third quartile

The ASD rate for children born to mothers with RA before delivery was estimated at 117 per 100,000 person-years, compared to 79 per 100,000 person-years for children born to mothers without RA. The crude HR was 1.70 (95% CI 1.07–2.69). After adjustment, this association was not statistically significant (HR = 1.55, 95% CI 0.98–2.46). The ASD rate for RA after delivery was estimated at 174 vs. 79 ASD per 100,000 person-years and the HRs were not statistically significant (crude HR = 1.91, 95% CI 0.86–4.24; adjusted HR = 1.75, 95% CI 0.79–3.88) (Table 2). RA before vs. RA after delivery was estimated at crude HR = 1.16 (95% CI 0.43- 3.12) and adjusted HR = 1.18 (95% CI 0.44–3.17). For the sensitivity analysis, the association between mothers with unspecified RA timing (before or after delivery) and ASD in the offspring was estimated at 127 vs. 79 ASD per 100,000 person-years. The crude HR was estimated at 1.60 (95% CI 1.07–2.38), while the adjusted HR was estimated at 1.40 (95% CI 1.02–1.91) (eTable 2).

**Table 2:** Hazard ratios for the risk of autism spectrum disorders (ASD) among offspring to mothers with rheumatoid arthritis (RA) compared to offspring to mothers without ra by cohort and pooled

Maternal RA status	Cohort - Health provider		ASD Rate	ASD Cases	Model1	Model2
			(per 100,000 person years)	(person years)	HR(95% CI)	HR(95% CI)
RA before delivery	Cohort I	RA	117	20 (17,103)	1.70 (1.07–2.69)	1.55 (0.98–2.46)
		Non-RA	79	1,919 (2,439,626)	Reference	Reference
	Cohort II	RA	146	33 (22,545)	1.92 (1.36 - 2.71)	1.83 (1.30 - 2.58)
		Non-RA	87	2,975 (3,404,054)	Reference	Reference
	Pooled	RA	134	53 (39,648)	1.87 (1.53 - 2.30)	1.77 (1.45 - 2.17)
		Non-RA	84	4,894 (5,843,680)	Reference	Reference
RA after Delivery	Cohort I	RA	174	6 (3,448)	1.91 (0.86–4.24)	1.75 (0.79–3.88)
		Non-RA	79	1,933 (2,453,281)	Reference	Reference
	Cohort II	RA	67	7 (10,400)	0.70 (0.33 - 1.48)	0.66 (0.31 - 1.38)
		Non-RA	88	3,001 (3,416,199)	Reference	Reference
	Pooled	RA	94	13 (13,848)	0.93 (0.61 - 1.41)	0.87 (0.57 - 1.32)
		Non-RA	84	4,934 (5,869,480)	Reference	Reference

1. RA: Rheumatoid arthritis; ASD: Autism Spectrum Disorder; reference: The comparison group; HR(95% CI): Hazard Ratio with 95% Confidence interval

3. ASD rate/100,000 person years = ASD cases/(sum of person year/100,000)

4. Model1: Adjusted for birth year by natural cubic splines with df = 3

5. Model2: Additionally adjusted for sex, maternal age at delivery (by natural cubic splines)

6. RA before delivery: RA diagnosed before delivery or within 3 months after delivery; RA after delivery: RA diagnosed after three months after delivery; Non-RA at delivery: The reference group for RA before delivery was Non-RA at delivery; Non-RA at delivery: The reference group for RA after delivery was Non-RA after delivery

#### Maternal RA and offspring ASD: cohort II

Of the 327,218 children born between 2003 and 2014 recorded in Cohort II, we excluded the observations with invalid dates (208, 0.06%), invalid records with conflicting times/diagnoses before the age of one (9,944, 3.04%), and incomplete data on primary covariates (7,370, 2.25%). There was no support in the data contradicting the assumption of proportional hazards after inspection of weighted Schoenfeld residuals. Our analytic cohort comprised 309,696 children and 3,426,599 person-years of follow-up (eFig. 1). There were 33 (1.92%) ASD diagnoses among children born to mothers with RA before delivery, while there were 2,975 (0.97%) ASD diagnoses among children born to mothers free from RA before delivery. Based on summary statistics (Table 1), compared to children born to mothers without RA before delivery, there was a slightly higher proportion of males and a higher maternal age at delivery for children born to mothers with RA during pregnancy.

The ASD rate for offspring of mothers with RA before compared with after delivery was estimated at 146 vs. 87 ASD per 100,000 person-years. The crude HR was estimated at 1.92 (95% CI 1.36–2.71); and the adjusted HR was estimated at 1.83 (95% CI 1.30–2.58). The HRs were not statistically significant for RA after delivery (crude HR = 0.70, 95% CI 0.33- 1.48; adjusted HR = 0.66, 95% CI 0.31–1.38; 67 vs. 88 ASD per 100,000 person-years) (Table 2). A direct comparison of the risk of ASD in the offspring of mothers with RA before and after delivery revealed an estimated rate of 146 vs. 67 ASD per 100,000 person-years. The crude hazard ratio (HR) was estimated at 2.66 (95% CI 1.13–6.22), while the adjusted HR was estimated at 2.74 (95% CI 1.19–6.34). In the context of the sensitivity analysis, the correlation between mothers with unspecified rheumatoid arthritis (before or after delivery) and autism spectrum disorder (ASD) in the offspring was estimated at a rate of 121 vs. 88 ASD per 100,000 person-years. The risk was estimated at an adjusted HR of 1.40 (95% CI 1.02–1.91) (eTable 2).

#### Maternal RA and offspring ASD: pooled estimates of two cohorts

The ASD rate for offspring to mothers with RA diagnosed before delivery was estimated at 134 vs. 84 ASD per 100,000 person-years for offspring born to mothers without an RA diagnosis at delivery. A diagnosis of maternal RA prior to delivery was associated with an increased risk of ASD in the offspring (pooled cohort HR = 1.87, 95% CI = 1.53–2.30). The risk of ASD in offspring was found to be statistically non-significant in cases where maternal RA was diagnosed after delivery (pooled cohort HR = 0.93, 95% CI = 0.61–2.30) (Table 2). In the context of the sensitivity analysis, the ASD rate for offspring to mothers with RA diagnosed before delivery was estimated at 123 vs. 84 ASD per 100,000 person-years. The risk of offspring ASD born to mothers with unspecified RA (before or after delivery) compared to unexposed offspring was estimated at an adjusted HR of 1.44 (95% CI 1.20–1.73) (eTable 2).

### Study 2: meta-analysis

We examined a total of 266 studies, of which 13 studies met the study inclusion criteria (eFigure 2, eTable 3–4) [17–20, 33–41]. Data quality was assessed by the Newcastle-Ottawa Scale for cohort studies (eTable 5) and case-control studies (eTable 6). Of the 8 cohort studies, all were considered of acceptable or good quality, with only two studies scored as low as 7. Of the 6 case-control studies, two studies were considered of somewhat lower quality, reaching the values of 6. The studies included in the meta-analysis were considered to have a low risk of bias. Of the 13 studies, three Danish studies [17, 20, 35] had overlapping study populations, and the largest Danish cohort was retained for meta-analysis [20]. Four Taiwanese studies [19, 33, 37, 39] had overlapping populations from the same cohort, and only one study was retained for the analysis of maternal RA before delivery [19] and another for sensitivity analysis of maternal RA unspecified (before or after delivery) [39]. We excluded three studies acking either crude or adjusted estimates [18, 36, 39] and another three that did not specify the timing of RA relative to delivery (eFigure 2) [20, 38, 41].

**Fig. 1:**
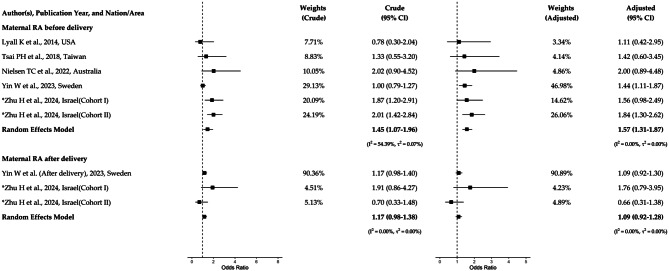
The odds ratios of autism spectrum disorders (ASD) in offspring with rheumatoid arthritis (RA) using random effects meta-analysis

The final dataset, together with two Israeli cohorts, comprised nine risk estimates (three with RA diagnosed before delivery and six after delivery) representative of 4,015,055 children of which 45,124 (1.1%) were with ASD. Two studies [20, 41], including the large Danish cohort study [20], had not specified the timing of RA and were therefore included in the sensitivity meta-analysis. With adjustments, the pooled OR was 1.57 (95% CI 1.31–1.87) for maternal RA before delivery, and 1.09 (95% CI 0.92–1.28) for maternal RA after delivery. Except for the crude OR of maternal RA before delivery, no evidence of heterogeneity was observed based on I-squared and tau-squared statistics (Fig. 1), and publication bias was not evident from the funnel plots (eFigure 3). Influence analyses suggested that no single study resulted in heterogeneity (eFigure 4–5). For the sensitivity analysis on studies without specifying the timing of maternal RA, the adjusted OR was estimated at 1.32 (95% CI 1.14–1.52) and was statistically significant.

## Discussion

The current study results, based on two birth cohorts and a meta-analysis, indicate that maternal RA diagnosed before but not after delivery is associated with an increased risk of ASD in the offspring. The observational study makes use of the strengths of data from two Israeli HMOs that provided clinically assessed, prospectively collected diagnoses of RA and ASD. The meta-analysis supported the association between maternal RA before delivery and the risk of offspring ASD.

Few cohort studies have separately investigated the association between the timing of maternal RA and offspring ASD (Fig. 1). Our pooled analysis, which shows an increased risk of ASD in children born to mothers with RA before delivery but not after, aligns with this Swedish study. In Cohort I of our study, the adjusted risk of ASD for maternal RA before delivery was not statistically significant and had wide confidence intervals. However, in Cohort II, as well as in the pooled analysis, the association reached statistical significance. This suggests that the null associations reported in other studies [19, 34, 37–39, 41] may be due to insufficient statistical power. Additionally, we observed an increased risk of ASD in children exposed to maternal RA of unspecified timing, with lower risk estimates compared to the risk in cases of maternal RA before delivery, consistent with results from a Danish cohort study using mixed timing of RA exposure.

The observed pattern where maternal RA before delivery was associated with offspring ASD, but RA occurring after delivery was not, may provide crucial insights into the underlying mechanisms. This suggests that ASD risk is associated with RA exposure specific to pregnancy and argues against a shared genetic etiology as a primary explanation. If common genetic factors were the predominant source of risk, i.e. pleiotropy, we would expect similar associations regardless of the timing of maternal RA onset relative to delivery. Future research, such as examining the co-occurrence of ASD and RA within individuals or employing family-based designs comparing sibling pairs with varying genetic correlation, may provide a more definitive answer about a shared genetic etiology. Future studies could also examine the role of maternal inflammatory status during pregnancy to better understand the underlying causal factors explaining offspring ASD risk. Earlier studies suggest a potential pathway through mother-to-child autoantibody transmission, including rheumatoid factor and antibodies against post-translational modified proteins such as citrullination and carbamylation [42], as well as elevated inflammatory mediators in utero [43–45]. This aligns with evidence that maternal immune activation can disrupt fetal neurodevelopment through mechanisms such as altered kynurenine pathway metabolism [46]. Important translational questions include whether timely RA treatment with anti-inflammatory biologics [47] could reduce offspring ASD risk, and whether timing of RA onset (pre-pregnancy versus by trimester) identifies a critical developmental window.

The current study strengths include a large population-based cohort design with integral replication, prospective identification of maternal RA and ASD diagnosed by clinical specialists, and a long follow-up period. These factors strengthened the current study and reduced the potential of selection bias, as legislation in Israel prohibits such bias. A particular strength of this study was the ability to differentiate between pre-delivery and post-delivery maternal RA exposure. The two Israeli cohort studies partly addressed the limitations such as insufficient power and oversimplified exposure, and filled the gap in the literature on maternal RA relative to offspring delivery. Furthermore, the current meta-analysis represents an improvement upon the prior meta-analyses [21–23] by examining the timing of RA relative to delivery. This novel feature facilitated the consideration of the role of prenatal exposure. The meta-analysis provided a comprehensive overview of the evidence on the association between maternal RA and the risk of offspring ASD across a range of global locations, enhancing generalizability.

## Limitations

Due to the limitations of our study, caution is required when interpreting the results. First, limited to very few cases of seronegative RA compared to seropositive cases, RA subtype analysis was not feasible in the cohort studies. Second, our data did not include information on paternal RA. However, prior research has not found that paternal RA is associated with an increased risk of offspring ASD [20, 40]. Third, it is important to acknowledge that the inclusion of the exposure “RA after delivery” also encompasses future RA information after the diagnosis of ASD, which may introduce bias. Furthermore, setting a specific age-span limit to avoid late RA exposure (e.g., toward the end of a mother’s life, typically beyond reproductive age) could have mitigated bias associated with longevity that is unrelated to the impact of RA on ASD offspring risk). Nonetheless, “RA after delivery” aimed to serve the purpose of representing the lifelong impact of heritable factors and allowed for an objective comparison to previous studies that did not differentiate the timing of RA. Fourth, the association between RA drugs (i.e., disease‑modifying antirheumatic drugs [48]) and offspring ASD risk has not been established due to a lack of data. Hence, the association between maternal RA before childbirth and offspring ASD may be the result of confounding by treatment. Finally, it should be noted that the associations between maternal RA and offspring ASD do not necessarily imply causality in this observational study, yet a clinical trial of this association is not ethically possible.

## Conclusions

Drawing on the strengths of two parallel birth cohorts from Israel and supported by meta-analysis, the current study indicates a modest but robust increase in the risk of ASD among offspring of mothers diagnosed with RA before delivery, but not when diagnosed only after delivery. This temporal specificity argues against shared genetic etiology and points toward maternal inflammatory status during pregnancy as a causal factor in offspring ASD risk.

## Electronic supplementary material

Below is the link to the electronic supplementary material.

Supplementary Material 1

## Data Availability

Data cannot be shared publicly because of legal restrictions. Data are available from the Israeli HMOs.
