# Supplementary material for: Maternal rheumatoid arthritis and the risk of offspring autism spectrum disorder: two national birth cohorts and a meta-analysis
Source: Mol Autism. 2025 Dec 4;16:61. doi: 10.1186/s13229-025-00694-w (PMC12679756; doi:10.1186/s13229-025-00694-w)
Supplement: Supplementary file 1 — Supplementary Material 1 [file 13229_2025_694_MOESM1_ESM.docx]

**Supplement**

[**eTable 1:** International Classification of Diseases (ICD) codes used for identification of Rheumatoid Arthritis (RA), Autism Spectrum Disorder (ASD) 2](#_Toc164788041)

[**eTable 2:** Hazard Ratios for the risk of Autism Spectrum Disorders (ASD) among offspring to mothers with Rheumatoid Arthritis (RA) compared to offspring to mothers without RA by cohort and pooled 3](#_Toc164788042)

[**eTable 3:** Search terms for studies of maternal Rheumatoid Arthritis (RA) and offspring autism spectrum disorder (ASD) 4](#_Toc164788043)

[**eTable 4:** Characteristics of eligible studies 5](#_Toc164788044)

[**eTable 5:** Assessment of risk of bias with the Newcastle-Ottawa Scale (cohort studies) 10](#_Toc164788045)

[**eTable 6:** Assessment of risk of bias with the Newcastle-Ottawa Scale (case control studies) 11](#_Toc164788046)

[**eFigure 1:** Flow chart of the inclusion and exclusion of Israeli cohorts derived from HMO1 and HMO2 12](#_Toc164788047)

[**eFigure 2:** Flow chart of inclusion and exclusion of the literature of systematic review 13](#_Toc164788048)

[**eFigure 3:** Funnel plots of the odds ratios of Autism Spectrum Disorders (ASD) in offspring with Rheumatoid arthritis (RA) using random effects meta-analysis 14](#_Toc164788049)

[**eFigure 4:** Influence analysis of the odds ratios of Autism Spectrum Disorders (ASD) in offspring with Rheumatoid arthritis (RA) using random effects meta-analysis 15](#_Toc164788050)

[**eFigure 5:** Leave-one-out analysis of the odds ratios of Autism Spectrum Disorders (ASD) in offspring with Rheumatoid arthritis (RA) using random effects meta-analysis 16](#_Toc164788051)

[**eFigure 6:** The odds ratios of Autism Spectrum Disorders (ASD) in offspring with Rheumatoid arthritis (RA) using random effects meta-analysis, based on the RA without specifying the timing of diagnosis 17](#_Toc164788052)

# **eTable 1:** International Classification of Diseases (ICD) codes used for identification of Rheumatoid Arthritis (RA), Autism Spectrum Disorder (ASD)

| Diagnosis | ICD 10* | ICD 9 |
| --- | --- | --- |
| **Rheumatoid Arthritis** | NA | 714.0*, 714.1*, 714.2*, 714.8* |
| Seropositive RA | NA | 714.0*, 714.1*, 714.2* |
| Seronegative RA | NA | 714.8* |
| **Autism Spectrum Disorder** | F84.0, F84.1, F84.3, F84.5, F84.8, F84.9 | 299.0* |
|  |  |  |

Abbreviations. ICD 9: International Classification of Diseases, Ninth Revision; ICD-10 International Statistical Classification of Diseases and Related Health Problems, Tenth Revision; * represents linking to any suffix; N/A: not applicable.

# **eTable 2:** Hazard Ratios for the risk of Autism Spectrum Disorders (ASD) in offspring to mothers with Rheumatoid Arthritis (RA) before delivery or after delivery compared to offspring to mothers without RA, by cohort and pooled

| **Cohort - Health provider** | | ASD Rate | ASD Cases | Model1 | Model2 |
| --- | --- | --- | --- | --- | --- |
|  | | (per 100,000 person years) | (person years) | HR(95% CI) | HR(95% CI) |
| Cohort I | RA | 127 | 26 (20,550) | 1.74 (1.17-2.60) | 1.60 (1.07-2.38) |
|  | Non-RA | 79 | 1,913 (2,436,178) | Reference | Reference |
| Cohort II | RA | 121 | 40 (32,945) | 1.48 (1.08 - 2.02) | 1.40 (1.02 - 1.91) |
|  | Non-RA | 88 | 2,968 (3,393,654) | Reference | Reference |
| Pooled | RA | 123 | 66 (53,495) | 1.53 (1.27 - 1.84) | 1.44 (1.20 - 1.73) |
|  | Non-RA | 84 | 4,881 (5,829,832) | Reference | Reference |

1. Abbreviations. HR, Hazard Ratio. CI., 95% CIs are Wald 2-sided 95% CIs;

2. Reference: The comparison group;

3. ASD rate/100,000 person years = ASD cases/(sum of person year/100,000)

4. Model1: Adjusted for birth year by natural cubic splines with df = 3

5. Model2: Additionally adjusted for sex, maternal age at delivery (by natural cubic splines)

**eTable 3:** Search terms for studies included in the meta-analysis

| **Database** | **Step** | **Terms** | **Pubmed** | **Embase** | **Web of science** | **Medline** |
| --- | --- | --- | --- | --- | --- | --- |
| Pubmed | 1 | (Mother) OR (Maternal) OR (Pregnancy) | 1,393,036 | 1,336,670 | 1,037,797 | 1,364,508 |
|  | 2 | (Rheumatoid Arthritis) OR (Autoimmune) | 397,439 | 558,320 | 404,492 | 373,179 |
|  | 3 | (Familial) OR (Offspring) OR (Children) OR (Fetus) OR (Kid) | 4,709,075 | 2,201,141 | 2,660,843 | 2,056,320 |
|  | 4 | (ASD) OR (Autism) OR (Autism spectrum disorder) | 82,286 | 116,662 | 123,743 | 85,461 |
|  | 5 | #1 AND #2 | 12,526 | 17,665 | 9,833 | 11,096 |
|  | 6 | #3 AND #4 | 52,449 | 45,301 | 66,927 | 36,223 |
|  | 7 | #5 AND #6 | **146** | **170** | **207** | **99** |

*Search data: 07 Jun, 2023

# **eTable 4:** Eligible study characteristics

| Author (s) year | Cohort name (follow-up time) | Study design | Country/Area | Number of participants | ASD cases | Number of children with RA mothers (Case group VS Control group) | Ascertainment method of identifying ASD and RA | Exposure timing | Effect size | Corresponding adjustments | Whether or not including into the meta-analysis | Justification |
| --- | --- | --- | --- | --- | --- | --- | --- | --- | --- | --- | --- | --- |
| Croen LA et al. 2005 | Northern California Kaiser Permanente Medical Care Program, Cohort (Jan 1995 - June 1999) | Case-control study | USA | 2,502 | 407 | 1 VS 6 | ASD: ICD-9 299.0 RA: ICD-9 | Before delivery: In the period of 2 years preceding delivery through 2 years following delivery were identified from inpatient and outpatient databases | NA | NA | No | Eligible but no adjustment of potential confounding; No effect size; Still, the two by two table can be extracted from the table 3 |
| Mouridsen SE et al.  2007 | Case: the population of patients attending the departments of child psychiatry in the university hospitals of Copenhagen and Aarhus, Admission time (1960-1984) Control: Danish Central Persons Register | Case-control study | Denmark | 441 | 111 | 1 VS 6 | ASD&RA: ICD-8 and ICD-9 | RA unspecified (before or after delivery) | NA | NA | No | Eligible but potentially overlapping to the Rom 2018 cohort |
| Atladóttir HO et al.  2009 | Danish Civil Registration System, Cohort(Jan 01 1993 - Dec 31 2004) | Cohort study | Denmark | 689,196 | 3,325 | NA | ASD&RA: ICD-8 and ICD-10 | RA unspecified (before or after delivery) | IRR: 1.70 (1.07–2.54) | Incidence rate ratio (IRR) were adjusted for age and its interaction with gender, calendar year, place of birth, and ages of the mother and father at the time of the child's birth | No | Eligible but potentially overlapping to the Rom 2018 cohort |
| Lyall K et al.  2014 | CHildhood Autism Risk from Genetics and the Environment (CHARGE) study, Cohort (started from 2022) | Case-control study | USA | 951 | 560 | 9 VS 8 | ASD: Self-reported questionnaires RA: Self-reported questionnaires and available medical records | Before delivery: Before pregnancy, during pregnancy, or both | HR: 1.11 (0.42-2.97) | Adjusted for maternal age | Yes | Eligible and no overlapping |
| Tsai PH et al.  2018 | National Health Insurance database and National Birth Registry, Cohort (2001-2012) | Matched cohort | Taiwan | 1,893,244 | 10,631 | 673 | ASD&RA: a catastrophic illness certificate from clinicians | Before delivery: Earlier than the date of conception | HR: 1.42 (0.60–3.40) | Adjusted for maternal age, 1-minute Apgar score, 5-minute Apgar score, mode of delivery, sex of child, gestational age, birth weight and place of residence | Yes | Eligible and overlapping but selected since it was the latest and largest representative study population in Taiwan with the RA defined as before delivery |
| Croen LA et al. 2019 | Study to Explore Early Development (SEED), Case control study (2003-2006) | Case-control study | USA | 1,578 | 663 | 9 VS 6 | ASD: Self-reported questionnaires RA: computer-assisted telephone interview on paper forms/questionnaires | Before delivery: Prior to the child's delivery date | NA | NA | No | Eligible but no adjustment of potential confounding; No effect size but the two by two table can be extracted from the table 3 |
| Rom AL et al. 2018 | Danish national registries, Cohort (1977-2008) | Cohort study | Denmark | 1,917,723 | 8,985 | 13,556 | ASD: ICD-8 (299.00, 299.01, 299.02, 299.03) and ICD-10 (F84.0, F84.1, F84.5, F84.8, F84.9) RA: ICD-8 (ICD-8: 712.19, 712.39, 712.59) and ICD-10 M05 and M06 ([except M06.1 Stills Disease]) | After delivery: RA diagnosed before birth | HR: 1.39 (1.11-1.75) | Adjusted for maternal age, paternal age, parity, maternal education and paternal RA | Yes | Eligible and overlapping but selected since it was the latest and largest representative study population in Denmark |
|  |  |  |  |  |  |  |  | RA unspecified (before or after delivery) | HR: 1.31 (1.06-1.63) |  |  |  |
| Spann MN et al. 2019 | Finnish national cohort (1987-2007) | Nested case control study | Finland | 22,658 | 4,600 | 51 VS 175 | ASD: 1CD-9 (299×) and ICD-10 (F84×)  RA: ICD-8:712, ICD-9:714; ICD-10: M05, M06 | RA unspecified (before or after delivery) | OR: 1.1  (0.8-1.5) | Adjusted for SES, gestational age, weight for gestational age, maternal age, and maternal psychiatric diagnosis | Yes | Eligible and no overlapping |
| Lee H et al.  2021 | Taiwan's National Health Insurance, Cohort (Jan 2001 -Dec 2008) | Cohort study | Taiwan | 708,517 | 4,506 | 231 | ASD: ICD-9 (299) RA: ICD-9 (714.0) | Before delivery: From January 1 1996 to the childbirth | HR: 1.38 (0.35–5.53) | Adjusting for family demographic data (income level and residence), maternal ages, maternal mental disorders, and sex of children | No | Eligible but potentially overlapping to the Tsai 2018 |
| Nielsen TC et al.  2022 | New South Wales (NSW) Perinatal Data Collection, Cohort (Jan 2002 - Dec 2008) | Case-control study | Australia | 55,353 | 24 | NA | ASD: ICD-10 (F84)/disability service report RA: ICD-10 (M05, M06) | RA unspecified (before or after delivery) | HR: 2.00 (0.89–4.48) | Adjusted for child sex | Yes | Eligible and no overlapping |
| Li DJ et al.  2022 | Taiwan’s National Health Insurance Research Database, Cohort (2004-2016) | Cohort study | Taiwan | 1,386,260 | 13,885 | 9,074 | ASD: ICD-9 (299) and ICD-10 (F84)  RA: ICD-9 (714) and ICD-10 (M05, M08, M45) | RA unspecified (before or after delivery) | HR: 1.13 (0.94–1.37) | Adjusted for individual and relative’s nonlinear age effects modelled by the restricted cubic spline function in the Cox regression | Yes | Eligable and overlapping but selected since it was the latest and largest representative study population in Taiwan with the RA defined as RA unspecified (before or after delivery) retrospective RA |
| Chiu HJ et al.  2022 | Taiwan's National Health Insurance, Cohort (2001-2010) | Matched cohort | Taiwan | 263,791 | 297 | 18,700 | ASD: ICD-9 (299) RA: ICD-9 (714.0) | RA unspecified (before or after delivery) | HR: 1.49 (1.01–2.20) | Adjusted for demographic data, prevalence of parental mental disorders and offspring RA | No | Eligible but potentially overlapping to the Li 2022 |
| Yin W et al.  2023 | Swedish national cohort (1995-2015) | Cohort study | Sweden | 1,503,908 | 28,962 | 3,629 | ASD: ICD10 (F840, F841, F843,  F845, F848, F849)  RA:ICD-10 (M05, M06)  ICD-9 (714A, 714B,  714C, 714W) | Before delivery | HR: 1.43 (1.11-1.84) | Adjusted for maternal and paternal age, socioeconomic status, and psychiatric history | Yes | Eligible and no overlapping |
|  |  |  |  |  |  |  |  | After delivery | HR:1.17 (0.98-1.40) |  |  |  |
| Zhu H et al. 2024  (Cohorts of this study) | Israeli national cohorts (2003-2014) | Cohort study | Israel | Cohort I = 251,903  Cohort II = 309,696 | Cohort I = 1,939  Cohort II = 3,008 | Cohort I = 1,408  Cohort II = 1,717 | ASD: ICD10 (F84.0, F84.1, F84.3, F84.5, F84.8, F84.9 299.0*)  RA: ICD9 (714.0*, 714.1*, 714.2*, 714.8*) | Before delivery | Pooled HR: 1.77 (1.45 - 2.17) | Adjusted for sex and maternal age at delivery | Yes | Eligible and no overlapping |
|  |  |  |  |  |  |  |  | After delivery | Pooled HR: 0.87 (0.57 - 1.32) |  |  |  |

# **eTable 5:** Assessment of risk of bias with the Newcastle-Ottawa Scale (cohort studies)

|  | Selection | | | | Comparability | | Outcome | | | Whether or not including into the meta-analysis | Total |
| --- | --- | --- | --- | --- | --- | --- | --- | --- | --- | --- | --- |
| Author (s), Year | Representativeness of the exposed cohort | Selection of the non-exposed cohort | Ascertainment of exposure | Outcome of interest not present at start of the study | Control for primary confounders | Control for  secondary  confounders | Assessment of outcome | Duration of follow-up | Adequacy of follow-up |  |  |
| Atladóttir HO et al. (2009) | 1 | 1 | 1 | 1 | 1 | 1 | 1 | 1 | 1 | No | 9 |
| Tsai PH et al. (2018) | 1 | 1 | 1 | 1 | 0 | 1 | 1 | 1 | 1 | Yes | 8 |
| Rom AL et al. (2018) | 0 | 0 | 1 | 1 | 1 | 1 | 1 | 1 | 1 | Yes | 7 |
| Lee H et al. (2021) | 1 | 1 | 1 | 1 | 0 | 1 | 1 | 1 | 1 | No | 8 |
| Li DJ et al. (2022) | 1 | 1 | 1 | 1 | 1 | 1 | 1 | 1 | 1 | No | 9 |
| Chiu HJ et al. (2022) | 1 | 1 | 1 | 1 | 1 | 1 | 1 | 0 | 0 | No | 7 |
| Yin W et al. (2023) | 1 | 1 | 1 | 1 | 1 | 1 | 1 | 1 | 1 | Yes | 9 |
| Zhu H et al. (2024)  (Cohorts of this study) | 1 | 1 | 1 | 1 | 1 | 1 | 1 | 1 | 1 | Yes | 9 |

* Representativeness of the exposed cohort: 1 point awarded if the exposed cohort is truly representative of the average cases of maternal RA in the community; Selection of the non-exposed cohort: 1 point awarded if drawn from the same community as the exposed cohort; Ascertainment of exposure: 1 point awarded if the maternal RA was derived from secure record or structure interview; Outcome of interest not present at start of the study: 1 point awarded if children with ASD at baseline were excluded; Control for primary confounders: 1 point awarded if adjustment for age/ birth year, and sex; Control for secondary confounders: 1 point awarded if adjustment for any additional factor; Assessment of outcome: 1 point awarded if children alive were confirmed by with ASD diagnosis or free from ASD, and the death was confirmed by death certificates; Duration of follow-up; 1 point awarded if follow-up ≥ 1 year; Adequacy of follow-up: 1 point awarded if all subjects accounted for complete follow-up.

# **eTable 6:** Assessment of risk of bias with the Newcastle-Ottawa Scale (case control studies)

|  | Selection | | | | Comparability | | Exposure | | | Whether or not including into the meta-analysis | Total |
| --- | --- | --- | --- | --- | --- | --- | --- | --- | --- | --- | --- |
| Author (s), Year | Is the case definition adequate? | Representativeness of the cases | Selection of Controls | Definition of Controls | Factor which study controls for | Study controls for any additional factor | Ascertainment of exposure | Same method of ascertainment for cases and controls | Non-Response rate |  |  |
| Croen LA et al. 2005 | 1 | 1 | 1 | 1 | 1 | 1 | 1 | 1 | 1 | No | 9 |
| Mouridsen SE et al. 2007 | 1 | 1 | 1 | 1 | 0 | 0 | 0 | 1 | 1 | No | 6 |
| Lyall K et al. 2014 | 0 | 1 | 1 | 1 | 1 | 1 | 0 | 1 | 1 | Yes | 7 |
| Croen LA et al. 2019 | 0 | 1 | 0 | 1 | 1 | 1 | 0 | 1 | 1 | No | 6 |
| Spann MN et al. 2019 | 1 | 1 | 1 | 1 | 1 | 1 | 1 | 1 | 1 | Yes | 9 |
| Nielsen TC et al. 2022 | 1 | 1 | 1 | 1 | 1 | 1 | 1 | 1 | 1 | Yes | 9 |

* Is the case definition adequate?: 1 point awarded if the ASD case has independent validation; Representativeness of the cases: 1 point awarded if the cases were consecutive or obviously representative series of cases; Selection of Controls: 1 point awarded if the controls were community controls; Definition of Controls: 1 point awarded if the control was described “had no history of ASD”; Factor which study controls for: 1 point awarded if adjustment for age/ birth year, and sex; Study controls for any additional factor: 1 point awarded if adjustment for any additional factor; Ascertainment of exposure: 1 point awarded if the diagnosis of RA was from secure record or structured interview where bind to case/control status; Ascertainment of exposure: 1 point awarded if same method was applied to ASD cases; Non-Response rate: 1 point awarded if the rate was the same for both groups.

# **eFigure 1:** Flow chart of the inclusion and exclusion of Israeli cohorts derived from HMO1 and HMO2


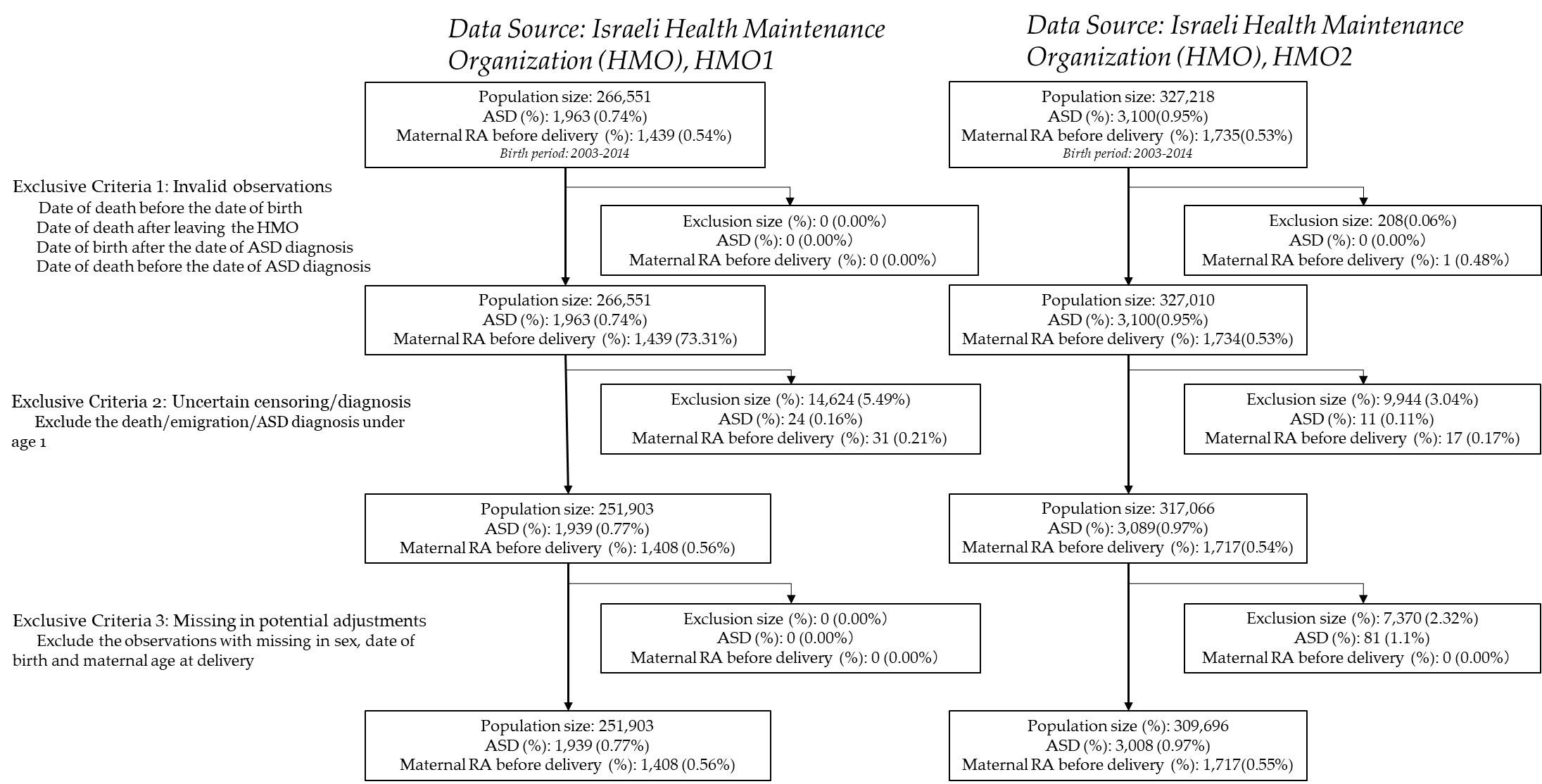


# **eFigure 2:** Flow chart of inclusion and exclusion of the literature of systematic review


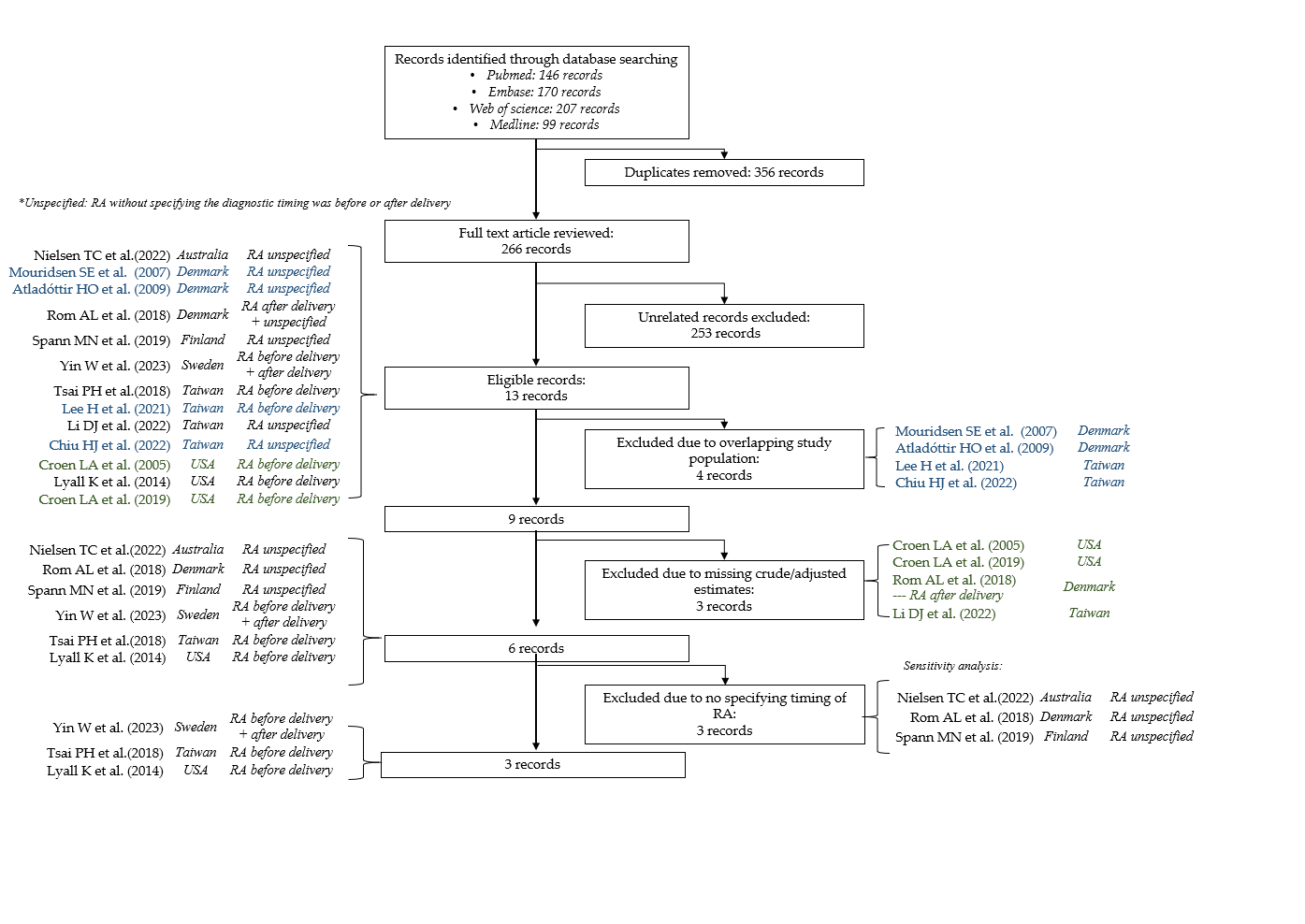


# **eFigure 3:** Funnel plots of the odds ratios of Autism Spectrum Disorders (ASD) in offspring with Rheumatoid arthritis (RA) using random effects meta-analysis


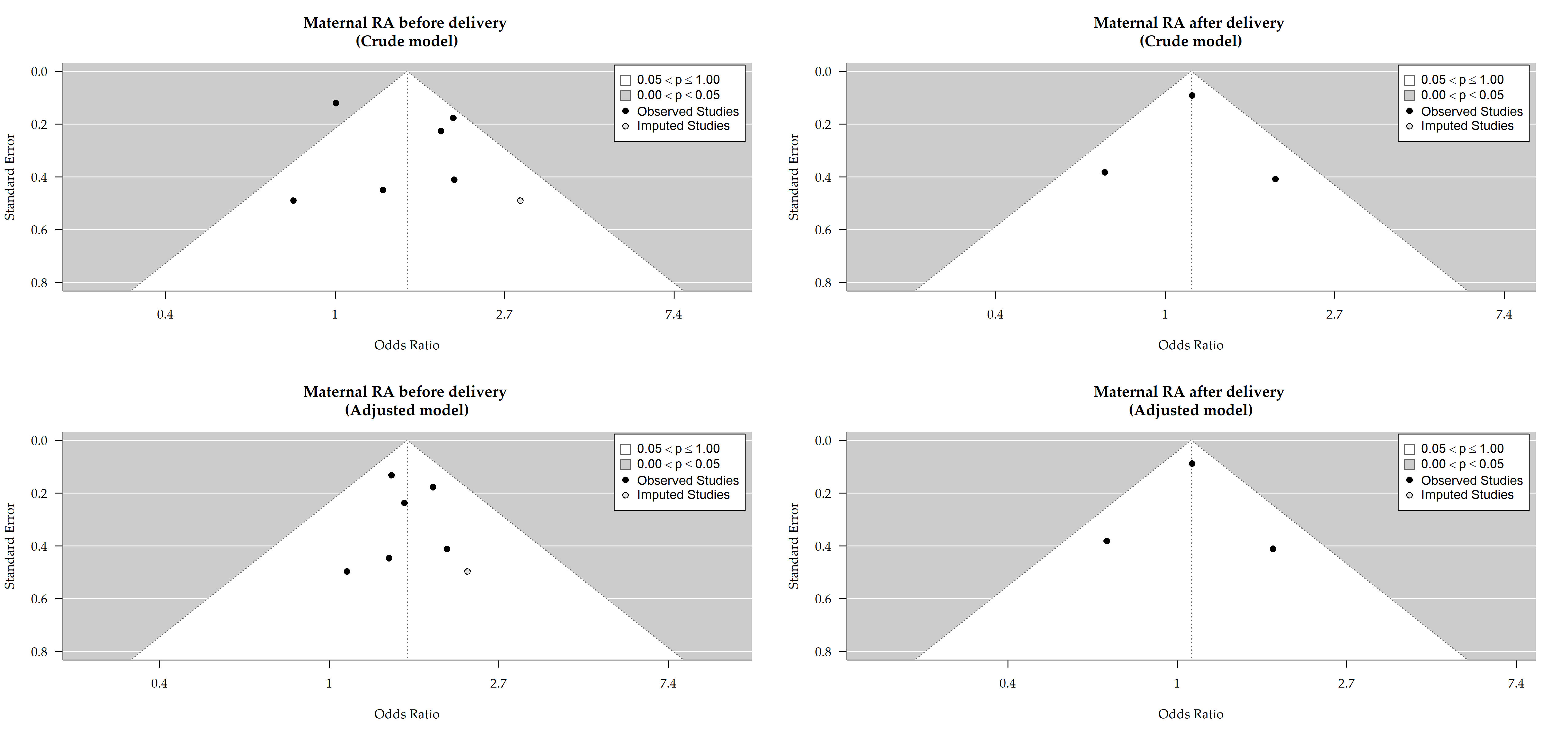


This figure showed the funnel plots of crude models and adjusted model using “RA before delivery” and “RA after delivery definitions, and in the meta-analysis. X-axis represented the odds ratio (OR); Y-axis showed standard error of the (OR). Publication bias was assessed by the degree of asymmetry where an asymmetric plot is indicative of publication bias

# **eFigure 4:** Influence analysis of the odds ratios of Autism Spectrum Disorders (ASD) in offspring with Rheumatoid arthritis (RA) using random effects meta-analysis


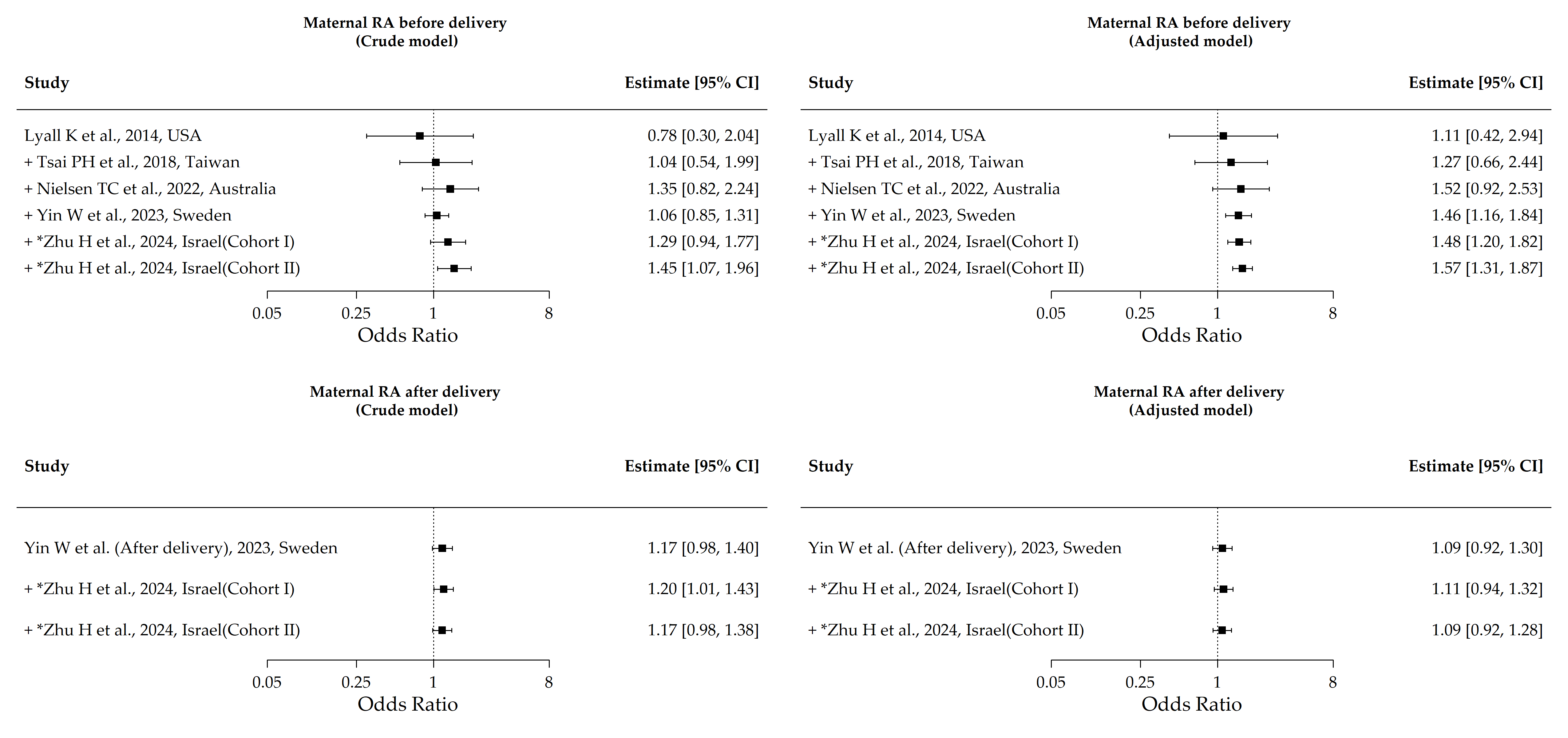


Influence analysis added one study at a time to test whether a single study had substantial effect in causing heterogeneity. Hollow squares represent individual estimate effects and solid lines represent 95% confidence intervals.

# **eFigure 5:** Leave-one-out analysis of the odds ratios of Autism Spectrum Disorders (ASD) in offspring with Rheumatoid arthritis (RA) using random effects meta-analysis


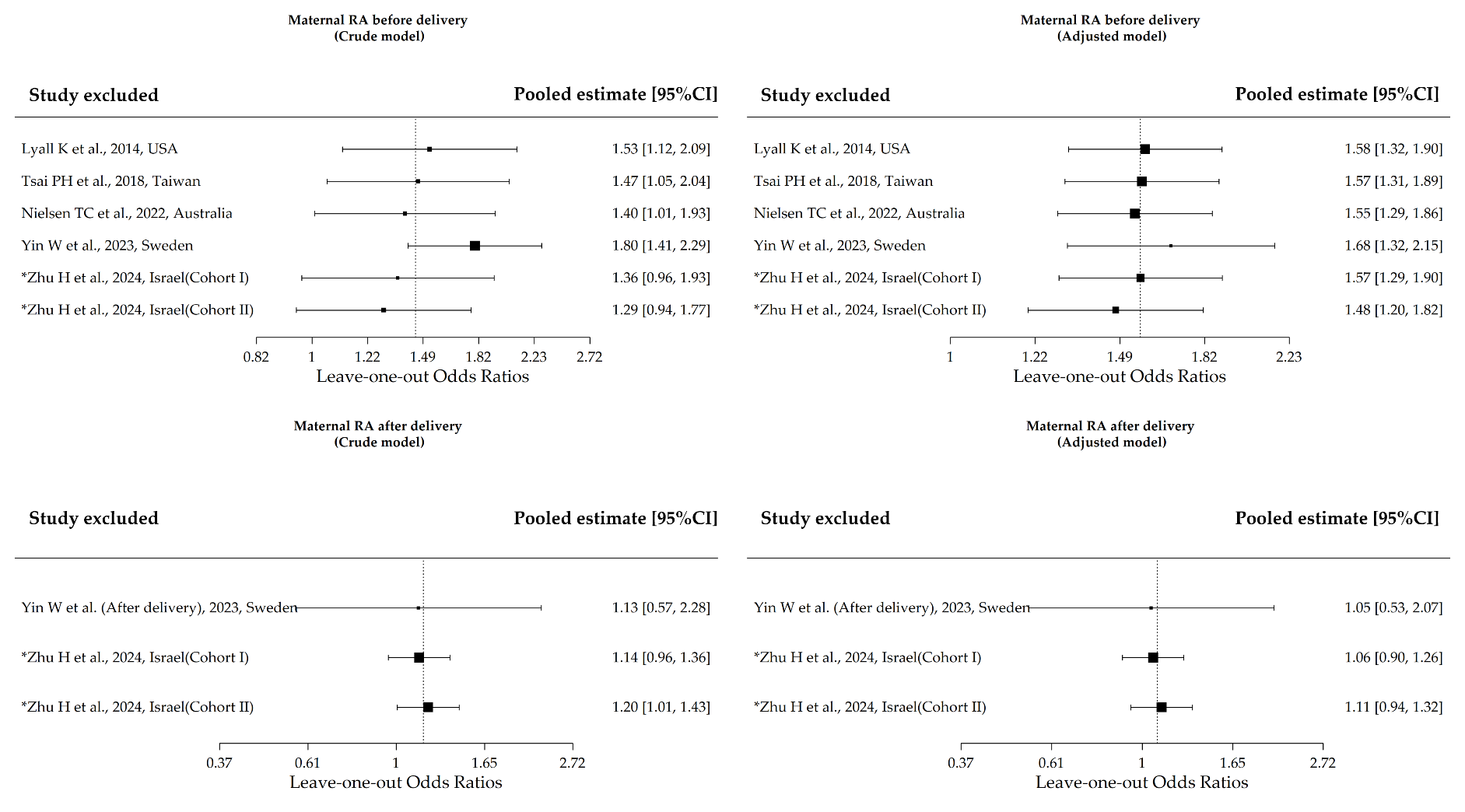


Leave-one-out analysis repeatedly excluded one study each time to test whether a single study has a substantial effect of causing heterogeneity. The left -hand side shows the study excluded in each leave—one-out analysis; the pooled estimate shows the pooled OR of the rest studies. The dashed line is the reference line of initial odds ratios. Hollow squares represent individual estimate effects and solid lines represent 95% confidence intervals. The size of the square represents the z-test statistics.

# **eFigure 6:** The odds ratios of Autism Spectrum Disorders (ASD) in offspring with Rheumatoid arthritis (RA) using random effects meta-analysis, based on the RA without specifying the timing of diagnosis


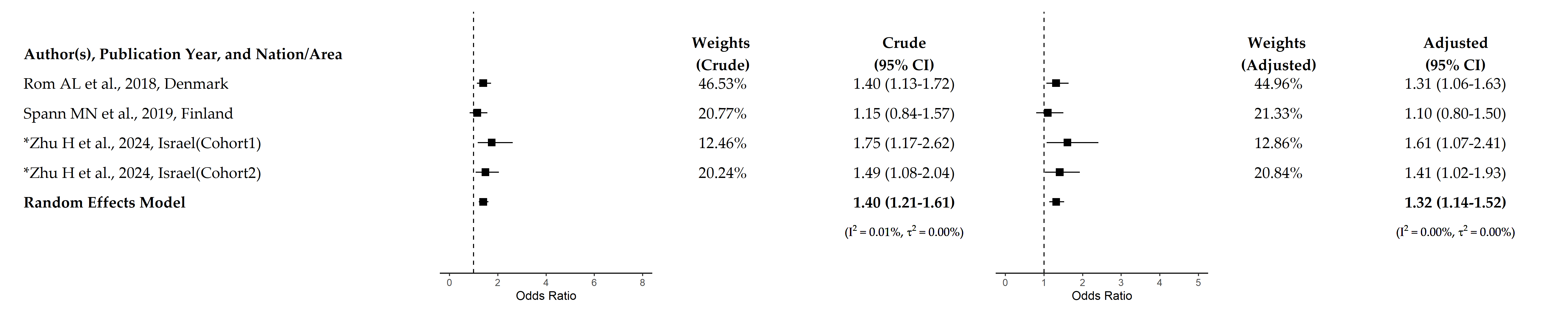


This figure showed the forest plot using the exposure of maternal RA without specifying the timing of diagnosis.
